# Genetic and Physiological Adaptations of Marine Bacterium *Pseudomonas stutzeri* 273 to Mercury Stress

**DOI:** 10.3389/fmicb.2018.00682

**Published:** 2018-04-05

**Authors:** Rikuan Zheng, Shimei Wu, Ning Ma, Chaomin Sun

**Affiliations:** ^1^Key Laboratory of Experimental Marine Biology, Institute of Oceanology, Chinese Academy of Sciences, Qingdao, China; ^2^Laboratory for Marine Biology and Biotechnology, Qingdao National Laboratory for Marine Science and Technology, Qingdao, China; ^3^College of Earth Sciences, University of Chinese Academy of Sciences, Beijing, China; ^4^College of Life Sciences, Qingdao University, Qingdao, China

**Keywords:** marine, *Pseudomonas stutzeri*, mercury, stress, motility, flagella

## Abstract

Mercury-mediated toxicity remains one of the greatest barriers against microbial survival, even though bacterial resistance to mercury compounds can occur. However, the genetic and physiological adaptations of bacteria to mercury stress still remains unclear. Here, we show that the marine bacterium *Pseudomonas stutzeri* 273 is resistant to 50 μM Hg^2+^ and removes up to 94% Hg^2+^ from culture. Using gene homologous recombination and complementation, we show that genes encoding Hg^2+^-transport proteins MerT, MerP, the mercuric reductase MerA and the regulatory protein MerD are essential for bacterial mercuric resistance when challenged with Hg^2+^. Further, mercury stress inhibits flagellar development, motility, chemotaxis and biofilm formation of *P. stutzeri* 273, which are verified by transcriptomic and physiological analyses. Surprisingly, we discover that MerF, a previously reported Hg^2+^-transporter, determines flagellar development, motility and biofilm formation in *P. stutzeri* 273 by genetic and physiological analyses. Our results strongly indicate that MerF plays an integral role in *P. stutzeri* 273 to develop physiological responses to mercury stress. Notably, MerF homologs are also prevalent in different human pathogens. Using this unique target may provide novel strategies to control these pathogenic bacteria, given the role of MerF in flagella and biofilm formation. In summary, our data provide an original report on MerF in bacterial physiological development and suggest that the *mer* in marine bacteria has evolved through progressive, sequential recruitment of novel functions over time.

## Introduction

Mercury, as one of the most toxic heavy metals naturally present in the earth, endangers the environment and causes a variety of diseases in humans and animals (Dash and Das, 2012). Specifically, exposure to mercury stress can dramatically decrease reproduction like fertilization capability, hatchability, viability and sperm motility (Dietrich et al., 2010). In the aquatic environment including the ocean, increasing evidence indicates that sublethal mercury pollution may cause a long-term decline and eventual extinction of species by adversely affecting fertilization and limiting propagation of a species (Kime, 1995).

Since mercury is ubiquitous in the earth, microorganisms inevitably encounter this heavy metal in their natural environment (Singh et al., 2014). Responding to environmental changes is a fundamental property of unicellular organisms who directly interact with an ever-changing microenvironment (Singh et al., 2014). Mercuric effects on live microorganisms impact their physiology (Dietrich et al., 2010). Through evolution, motility and chemotaxis are remarkably evolved features of bacterial physiology and strengthen bacterial adaptive capabilities to mercuric stress (Gadd, 2010). Chemotaxis of a bacterium toward metal stress largely depends on its physiological capability to utilize or resist the metallic environment or bacterial swarming powered by rotating helical flagella, a universal movement patterns (Kearns, 2010). In addition, bacteria within a developing biofilm may require chemotaxis and/or motility to move along the surface, thereby facilitating the growth and spread of the biofilm (Stelmack et al., 1999). With extensive evolution, mercury-resistant bacteria obtain the *mer* (mercuric ion resistance) operon in their genome to respond to stress from toxic mercury compounds. The *mer* operon enables bacteria to survive in the presence of mercury and reduce it to volatile, less-toxic Hg^0^, which diffuses out of the cell (Mathema et al., 2011).

Of the known bacterial heavy metal resistance systems, the *mer* operon is an intensively studied mercurial resistance system. Typically, the *mer* operon consists of a series of structural genes that transport and transform inorganic and organic mercury, such as mercuric reductase (MerA), organomercury lyase (MerB), periplasmic Hg^2+^ scavenging protein (MerP), one or more inner membrane spanning proteins (MerT, MerC, MerE, MerF, and MerG) that transport Hg^2+^ to the cytoplasm for reduction by MerA and regulatory proteins (MerR, MerD). Expression of the *mer* operon is tightly regulated by the dual function transcriptional regulator, MerR, which binds to the *mer* operator/promoter (O/P) region and acts as a transcriptional repressor or activator in the absence or presence of Hg^2+^ (Mathema et al., 2011). MerD, the other proposed regulatory protein of *mer* operon, may function as either an activator (Nucifora et al., 1989) or a repressor of the *mer* operon (Mukhopadhyay et al., 1991). MerD can form a ternary complex in association with O/P region and MerR to co-regulate the expression of *mer* operon (Champier et al., 2004). Following exposure to ionic Hg^2+^, the toxic metal firstly binds to two cysteine residues at positions 14 and 17 of MerP (Steele and Opella, 1997), which directly transfers the Hg^2+^ to the mercury-specific transporter MerT (Schue et al., 2008). MerT transfers Hg^2+^ from its two cysteine residues located on the periplasmic side of the membrane to two cysteine residues located on the cytosolic side (Morby et al., 1995). Once bound on the cytosolic side of MerT, the Hg^2+^ is transferred directly to two cysteine residues within the amino-terminal domain of MerA (NMerA) (Schue et al., 2008) which then transfers the Hg^2+^ to two cysteine residues in the active site of MerA for NAD(P)H-dependent reduction to Hg^0^ (Hong et al., 2014). The mercuric reductase MerA is the central enzyme in the microbial mercury detoxification system and *merA* is a suitable biomarker for examining the functional diversity of Hg detoxification (Boyd and Barkay, 2012). The *merE* gene is a predicted small ORF immediately following *merD* in many Gram-negative *mer* operon sequences, and it is proposed to encode a transporter of Hg^2+^ or methylmercury (Sone et al., 2017). MerF could transport ionic mercury from the periplasmic protein MerP across the bilayer to MerA and it plays a similar function as that of MerT (Barkay et al., 2003). However, the other functions of MerF toward mercury resistance are still unclear given that MerT is considered as the main transporter of mercury (Wahba et al., 2017).

Despite being the most studied bacterial toxic metal resistance loci, significant issues remain unknown to elucidate the genetic and physiological adaptations of bacteria to mercury stress, such as the relationship between each gene in the *mer* operon and bacterial physiology toward mercury resistance. While the role of the *mer* operon is well understood in terrestrial bacteria, however, it is not conclusive how *mer* works to cope with mercury stress in marine bacteria (Barkay et al., 2003). Notably, increasingly serious mercuric pollution in the ocean not only leads to food chain contamination, but also hastens ocean acidification (Wang et al., 2017). So, we must quickly develop cost-effective, sustainable and environmentally friendly remediation methods that facilitate the removal of mercury from contaminated ocean sites (Sone et al., 2013). But, we can only develop novel bioremediation strategies with a comprehensive understanding how *mer* systems function in response to the ever-increasing challenges of co-existing with mercury.

In this study, we show that the mercury resistant marine bacterium *P. stutzeri* 273 contains a *mer* gene cluster consisting of putative regulatory proteins (MerR, MerD), transporters (MerT, MerP, MerE, and MerF) and the mercuric reductase (MerA). Using genetic and transcriptomic assays, we demonstrate that *merT*, *merA*, *merP, merD*, and *merR* confer mercury resistance in *P. stutzeri* 273. Importantly, we identify novel functions of MerF to promote bacterial mercury resistance, such as the determination of flagellar development, motility, chemotaxis and biofilm formation. Finally, we propose a model for the mercury-adapted lifestyle of the bacterium *P. stutzeri* 273.

## Materials and Methods

### Bacterial Strains and Media

*Pseudomonas stutzeri* 273 was isolated from the sediment samples collected by RV *KEXUE* during a cruise in the East China Sea in the year of 2014 (Wu et al., 2016). *P. stutzeri* 273 and its mutants were cultured in marine broth 2216E (5 g/L tryptone, 1 g/L yeast extract, one liter filtered seawater, pH adjusted to 7.4–7.6) or Luria Bertani (LB) medium (10 g/L peptone, 5 g/L yeast extract, 10 g/L NaCl, pH adjusted to 7.0) and incubated at 28°C under vigorous agitation at speed of 150 rpm. *Escherichia coli* DH5α was used as the host for plasmid construction, and *E. coli* S17-1 was used as a vector donor in conjugation. *E. coli* DH5α, *E. coli* SY327, and *E. coli* S17-1 were grown in LB medium at 37°C with shaking at speed of 150 rpm. When necessary, antibiotics were used at the following concentrations: chloramphenicol (Cm) and gentamicin (Gm) with a final concentration of 25 and 25 μg/mL, respectively (Wu et al., 2017).

### Bioinformatic Analysis

The sequences of genes within the *mer* gene cluster of *P. stutzeri* 273, MerD and MerF protein sequences of other bacteria used in our study were obtained from GenBank. All of the bacterial MerF sequences were aligned using ClustalW2 (Larkin et al., 2007), and the phylogenetic tree was constructed with MEGA6.0 (Tamura et al., 2013). The complete genome sequence of *P. stutzeri* 273 has been deposited at GenBank under the accession number CP015641, and all the genome information of *P. stutzeri* 273 available in GenBank.

### Determination of Mercury Minimal Inhibitory Concentrations (MIC), Growth Curves and Mercury Removal Rate in *P. stutzeri* 273 or *P. aeruginosa* PAO1

To determine the mercury MIC against *P. stutzeri* 273, fifty-microliter of mid-log-phase cultures was inoculated in 5 mL LB medium containing 0, 20, 40, 60, 80, 100, and 120 μM HgCl_2_, respectively. Cultures were incubated at 28°C with the speed of 150 rpm for 24 h for each strain tested until confluent growth was observed in control (no HgCl_2_) tubes, followed by examination of growth on the HgCl_2_-supplemented groups. The MIC value was the lowest concentration of Hg^2+^ at which growth in LB medium was inhibited (Vetriani et al., 2005). Each treatment was performed in triplicate and the MIC was defined as the average number of three repeats. To determine the mercury MIC against *P. aeruginosa* PAO1, fifty-microliter of mid-log-phase cultures was inoculated in 5 mL LB medium containing 0, 0.1, 0.2, 0.3, 0.4, 0.5, 0.6, 0.7, 0.8, 0.9, and 1.0 μM HgCl_2_, respectively. Other operations were performed as described above. To check the growth of *P. stutzeri* 273 under HgCl_2_ stress, 1 mL overnight bacterial culture was inoculated in 100 mL flask at 28°C with the speed at 150 rpm in LB medium in the absence or presence of 20 μM or 50 μM HgCl_2_. Bacterial growth status was monitored by measuring the OD_600_ value every 4 h until cell growth reached stationary phase. To determine the Hg^2+^ removal rate of *P. stutzeri* 273, *P. stutzeri* 273 was incubated at 28°C with speed of 150 rpm in LB medium supplemented with 20 μM or 50 μM HgCl_2_ to OD_600_ value of 1.5. The supernatant of culture was collected by centrifugation (13,400 × *g*, 2 min). After which, the supernatant was thoroughly digested with nitric acid and perchloric acid, and diluted with Milli-Q water for Hg^2+^ concentration detection. The dissolved Hg^2+^ concentrations were measured with an inductively coupled plasma source mass spectrometer (Optima 7300 DV, PerkinElmer) (Han et al., 2014).

### Construction of Deletion Mutants of Genes Within *mer* Gene Cluster and Complementation of Mutants for Mercury Sensitivity Assays

The *P. stutzeri* 273 derivatives (Δ*merA*, Δ*merP*, Δ*merR*, Δ*merT*, Δ*merD*, Δ*merE*, Δ*merF*, and Δ*fliC*) were constructed by allelic exchange as previously described (Wu et al., 2017). Briefly, fragments for mutant construction were amplified from the chromosome of *P. stutzeri* 273 by primers shown in Supplementary Table S1. Then, PCR fragments were purified, digested and ligated into the suicide vector pEX18Gm containing an *oriT* for conjugation. The resulting plasmid was transformed successively into *E. coli* SY327 and *E. coli* S17-1 by the CaCl_2_ method. Mating between *P. stutzeri* 273 and *E. coli* S17-1 containing different plasmids was performed at 30°C for 24 h. Colonies growing on LB agar amended with Cm (25 μg/mL) and Gm (25 μg/mL) were single-event positive recombinant strains. The individual colony was picked and incubated overnight at 30°C with shaking in LB broth with Cm (25 μg/mL) and Gm (25 μg/mL), then diluted 1:1000 into fresh LB broth and plated onto LB agar plate amended with 10% sucrose and incubated for 48 h at 30°C. A single colony was re-streaked several times before being replicated onto a LB agar plate amended with Gm (25 μg/mL) to confirm sensitivity to gentamicin and loss of the pEX18GM vector. All double recombination mutants candidates were verified by PCR amplification and sequencing.

To construct complementary strains, pUCP18 was used as the mother plasmid, and *E. coli* DH5a was used as the host strain. Briefly, genes (*merT*, *merP*, *merF*, *merA*, and *merD*) derived from *P. stutzeri* 273 together with their native promoters were amplified from the wild-type strain by primers listed in Supplementary Table S2. Then, the corresponding PCR products were digested with *Hin*dIII and *Bam*HI and ligated into pUCP18 to produce pUCP18-*merT*, pUCP18-*merP*, pUCP18-*meF*, pUCP18-*merA*, and pUCP18-*merD*, respectively. The above resulting plasmids were separately transformed into the corresponding mutants Δ*merT*, Δ*merP*, Δ*merF*, Δ*merA*, and Δ*merD* with the CaCl_2_ method (Lee et al., 2013; Weller-Stuart et al., 2017). The final complementary strains (Δ*merT/*c*merT*, Δ*merP*/c*merP*, Δ*merF*/c*merF*, Δ*merA*/c*merA*, and Δ*merD*/c*merD*) were verified by PCR amplification and sequencing.

Mercury sensitivity assays of *P. stutzeri* 273 wild type, different mutants and the corresponding complementations were performed on LB agar plates with different concentrations of HgCl_2_. Cells were grown to the exponential phase in LB liquid medium and then diluted to an OD_600_ of 0.1. Five 10-fold dilutions were carried out and spotted on agar plates incubated at 28°C for 48 h. Each experiment was repeated three times.

### Motility, Chemotaxis and Biofilm Formation Assays

For the motility assay, *P. stutzeri* 273 and correlative mutants were cultured in LB medium at 28°C with speed of 150 rpm to the OD_600_ of 1.2. Then 20 μL different culture was inoculated onto the center of 1% agar plate without or with 20 or 50 μM HgCl_2_ and incubated for 5 days at 28°C (Radin et al., 2013). For the chemotaxis assay, wild type *P. stutzeri* 273 was cultured in LB medium at 28°C with speed of 150 rpm to the OD_600_ of 1.2. Then 20 μL culture was inoculated onto the center of 1% agar plate, and the filter paper containing 20 μL water or 20 or 50 μM HgCl_2_ was loaded 2-cm away from the culture spot. Plates were incubated for 5 days at 28°C for further examination. For the biofilm formation assay, 50 μL of *P. stutzeri* 273 culture (OD_600_ about 0.8) was inoculated in a borosilicate tube containing 1 mL liquid LB medium without or with 20 and 50 μM HgCl_2_, respectively. The tubes were incubated at 28°C without shaking for 3 days for further biofilm assays. To quantify the amount of biofilm, the liquid LB medium and planktonic cells were removed from the borosilicate tubes, and the remaining biofilms were washed off with phosphate-buffered saline (PBS, pH 7.4) and stained with crystal violet. The stained biofilms were eluted in 100% ethanol and monitored for biofilm formation as determined by spectrophotometry at 595 nm (Lee et al., 2013). The biofilm formation assays for *P. stutzeri* 273 Δ*merF* and its complement were performed as described above.

### Western Blotting

Bacterial lysates of wild type *P. stutzeri* 273 and *merF* deletion mutant were harvested and lysed in the sample buffer containing sodium dodecyl sulphate (SDS) and β-mercaptoethanol, then boiled for 10 min at 100°C. Same amount proteins from wild type and mutant were separated on 12% SDS-page gels, electro-transferred to nitrocellulose membranes and incubated with FliC primary antibody (BioLegend) and secondary antibody (Proteintech Group).

### Quantitative Reverse Transcription-PCR (qRT-PCR)

For qRT-PCR, cells of *P. stutzeri* 273 or corresponding mutants incubated in LB medium amended without or with 20 or 50 μM Hg^2+^ to OD_600_ of 1.2. Cells were centrifuged at 6,000 × *g* for 10 min, total RNAs were extracted using the RNApure Bacteria Kit (DNase I) (CWBio, China). Total RNAs were reverse transcribed into cDNA, and the transcriptional levels of different genes were determined by qRT-PCR with Sybr Green Premix Low rox (MDbio, China) and the QuantStudioTM 6 Flex (Thermo Fisher Scientific, United States). RNA degradation was examined on 1% agarose gels. RNA purity was verified with NanoPhotometer R spectrophotometer (IMPLEN, Westlake Village, CA, United States). RNA concentration was determined by QubitR RNA Assay Kit in QubitR 2.0 Fluorometer (Life Technologies, Carlsbad, CA, United States). RNA integrity was assessed by RNA Nano6000 Assay Kit for the Bioanalyzer 2100 system (Agilent Technologies, Santa Clara, CA, United States). 16S rDNA was used as an internal reference. The relative gene expression was calculated using the 2^-ΔΔCt^ method with each transcript signal normalized to 16S rDNA (Petrus et al., 2015; Zhu et al., 2017). Transcript signals for each treatment were compared to the transcript signals from the control group. Specific primers for the genes within *mer* gene cluster and 16S rDNA were designed using Primer 5.0 as shown in Supplementary Table S3. All qRT-PCR runs were conducted with three biological and three technical replicates.

### Transcriptional Profiling of *P. stutzeri* 273 Challenged With Different Concentrations of Hg^2+^

Total RNAs of *P. stutzeri* 273 incubated in LB medium in the absence or presence of 20 or 50 μM of HgCl_2_ with OD_600_ about 1.2 were extracted and checked as described above. Detailed protocols of RNA-seq are described in the Supplementary Information. qRT-PCR verification of transcriptional profiling data was performed as described above. Five genes (PS273GM-RS08590, *flgG*; PS273GM-RS08570, *flgC*; PS273GM-RS08525, *motA*; PS273GM-RS02905, *cheY*; PS273GM-RS08565, *flgB*) were chosen for verification of transcriptional profiling data by qRT-PCR. The specific primers were listed in Supplementary Table S3. The heat map was made by HemI 1.0 based on the KEGG enrichment results.

### Transmission Electron Microscopy (TEM)

To examine the flagella formation, wild-type and relative mutants of *P. stutzeri* 273 were all examined using TEM with a JEOL JEM 12000 EX (equipped with a field emission gun) at 100 kV. The cell suspension was washed with sterile nutrient solution or Milli-Q water and centrifuged at 5,000 × *g* for 3 min. Finally, these samples were taken by immersing copper grids coated with a carbon film for 1 min in bacterial suspensions and washed for several minutes in distilled water and dried several minutes at room temperature (Bundeleva et al., 2014; Han et al., 2014).

### Statistical Analysis

Tests for significance of the differences among groups were subjected to one-way analysis of variance (one-way ANOVA) and multiple comparisons by using the GraphPad Prism 5. Statistical significance was defined in our study by *P* < 0.05 (indicated by^∗^ in all figures), *P* < 0.01 (indicated by ^∗∗^ in all figures) or *P* < 0.001 (indicated by ^∗∗∗^ in all figures).

## Results

### Characterization of Mercury Resistance Conferred by the *mer* Gene Cluster in *P. stutzeri* 273

**FIGURE 1:**
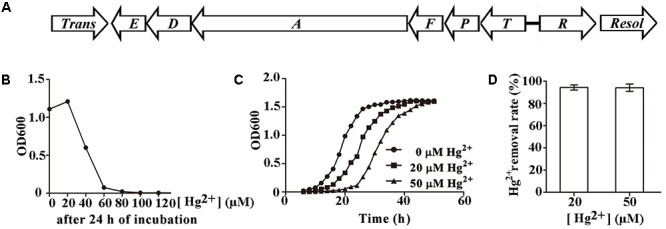
Characterization of mercury resistance by *Pseudomonas stutzeri* 273. **(A)** Arrangement of potential *mer* gene cluster in *P. stutzeri* 273. *Trans*, transposase (PS273GM_RS09950); *E*, *merE* (PS273GM_RS09955); *D*, *merD* (PS273GM_RS09960); *A*, *merA* (PS273GM_RS09965); *F*, *merF* (PS273GM_RS09970); *P*, *merP* (PS273GM_RS09975); *T*, *merT* (PS273GM_RS09980); *R*, *merR* (PS273GM_RS09985); *Resol*, resolvase (PS273GM_RS09990). Locus tags indicated in the bracket. **(B)** Hg^2+^ MIC determination in *P. stutzeri* 273. **(C)** Growth style of *P. stutzeri* 273 in the absence or presence of Hg^2+^ (20 μM or 50 μM). **(D)** Hg^2+^ removal by *P. stutzeri* 273 with different concentrations of Hg^2+^.

*Pseudomonas stutzeri* 273 has its entire genome sequenced (NCBI accession number CP015641) (Wu et al., 2017). When analyzing the genome sequence of this bacterium, a candidate *mer* gene cluster between nucleotide 2193317 to nucleotide 2196970 was found in the chromosome of *P. stutzeri* 273. Notably, the *mer* gene cluster is located on a genomic island flanked by a transposase (WP_046622512.1) and a resolvase (WP_003089068.1) (**Figure 1A**). This *mer* gene cluster consists of two potential mercury-responsive regulatory proteins, MerR and MerD, the mercury transporters MerP, MerT, MerE, and MerF, and the mercuric ion reductase MerA, which reduces toxic Hg^2+^ to non-toxic Hg^0^ (**Table 1**). To measure the potential mercury resistance conferred by the *mer* gene cluster, we examined the resistance of *P. stutzeri* 273 against Hg^2+^. Our results showed that Hg^2+^ has a 60 μM minimum inhibition concentration against *P. stutzeri* 273 (**Figure 1B**). Notably, the Hg^2+^ MIC against *P. aeruginosa* PAO1 without *mer* gene cluster in the chromosome is 0.7 μM (Supplementary Figure S1), which is much lower than that of *P. stutzeri* 273. The bacteria could grow to a similar density in the presence of 20 μM Hg^2+^ or 50 μM Hg^2+^ compared to growth with no mercury, however, the time reaching stationary phase is 10 and 20 h later than that without mercury stress, respectively (**Figure 1C**). We then assessed the mercury removal property of *P. stutzeri* 273 by measuring metal depletion in culture supernatants by inductively coupled plasma-optical emission spectroscopy (ICP-OES). We found that up to 94% Hg^2+^ could be removed in both 20 and 50 μM Hg^2+^ conditions (**Figure 1D**). Taken together, these results indicate that *P. stutzeri* 273 is a good candidate to develop bioremediation products toward mercuric pollution in the marine environment.

**Table 1:** Proteins involved in the mercury resistance of *Pseudomonas stutzeri* 273.

Protein designation	Size (amino acids)	Putative function
MerE	78	Methylmercury transport protein
MerD	121	Regulator protein
MerA	548	Mercuric ion reductase
MerF	81	Mercuric ion transport protein
MerP	91	Periplasmic mercuric ion binding protein
MerT	116	Mercuric ion transport protein
MerR	144	Regulator protein

### Genetic and Biochemical Determination of Key Genes in the *mer* Gene Cluster of *P. stutzeri* 273 Conferring Mercury Resistance

**FIGURE 2:**
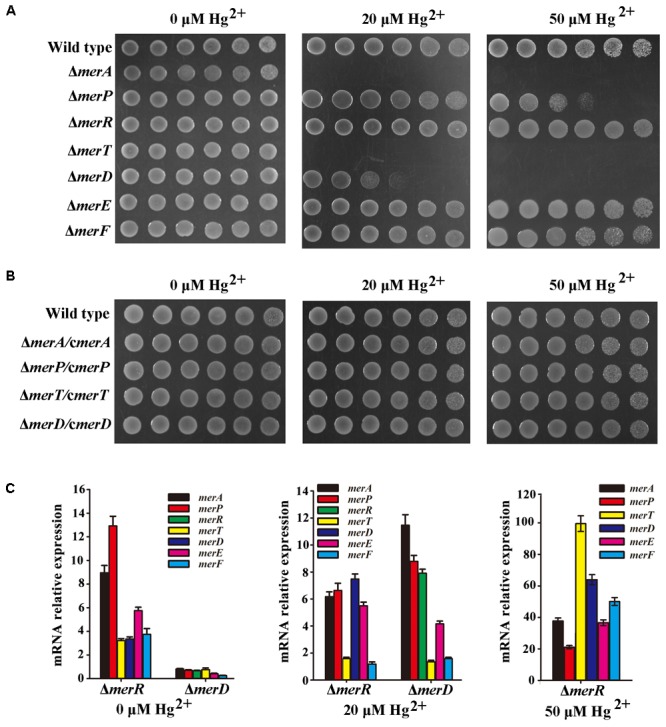
Genetic and biochemical analyses of *mer* gene cluster function for mercury resistance in *P. stutzeri* 273. **(A)** Growth in LB solid media of wild type, mutant strains (Δ*merA*, Δ*merP*, Δ*merR*, Δ*merT*, Δ*merD*, Δ*merE*, and Δ*merF*) and **(B)** complemented strains (Δ*merA/c*Δ*merA*, Δ*merP/c*Δ*merP*, Δ*merT/c*Δ*merT*, and Δ*merD/c*Δ*merD*) of *P. stutzeri* 273. Five 10-fold dilutions spotted from left to right with the indicated concentration of Hg^2+^. **(C)** Expression of each gene in the *mer* gene cluster of *P. stutzeri* 273 mutant with *merR* or *merD* deletion without Hg^2+^ (left panel), in the presence of 20 μM Hg^2+^ (middle panel). Expression of each gene in the *mer* gene cluster of *P. stutzeri* 273 mutant with *merR* deletion in the presence of 50 μM Hg^2+^ (right panel).

*Mer* gene cluster is widely accepted to determine mercurial resistance of most bacteria (Barkay et al., 2003). We sought to elucidate which genes in the *mer* gene cluster are essential for mercury resistance in *P. stutzeri* 273. So, we constructed different deletion mutants (Δ*merA*, Δ*merP*, Δ*merR*, Δ*merT*, Δ*merD*, Δ*merE*, Δ*merF*) and tested their growth status in LB agar plates supplemented with different concentrations of Hg^2+^. As expected, the wild type showed normal growth in the concentration of 50 μM Hg^2+^. However, the mutants with deletion of *merA* (encoding the mercuric reductase) or *merT* (encoding the Hg^2+^-transporter) exhibited the greatest sensitivity to the low concentration of Hg^2+^, such that the cells could not even grow in the agar plate containing 20 μM Hg^2+^ (**Figure 2A**, middle panel). The deletion of *merD* (encoding the regulatory protein of *mer* gene cluster) affected bacterial growth to a certain extent in the presence of 20 μM Hg^2+^ (**Figure 2A**, middle panel), but cellular growth was completely inhibited by 50 μM Hg^2+^ (**Figure 2A**, right panel). The deletion of *merP* (encoding the Hg^2+^-transporter) only affected bacterial growth under the 50 μM Hg^2+^ condition but not the low level Hg^2+^ condition (**Figure 2A**, middle and right panels). On the other hand, deletions of *merR* (encoding the regulatory protein of *mer* gene cluster), *merE* and *merF* (encoding Hg^2+^-transporter) did not affect the mercury resistance of *P. stutzeri* 273, as the cells grew as well as the wild type in the presence of 50 μM Hg^2+^ (**Figure 2A**, right panel). To better understand how *merA*, *merP*, *merT*, and *merD* confer mercury resistance to *P. stutzeri* 273, we constructed complements (Δ*merA*/c*merA*, Δ*merP*/c*merP*, Δ*merT*/c*merT*, and Δ*merD*/c*merD*) of the above deletion strains and examined their growth in the presence of both low (20 μM) and high (50 μM) levels of Hg^2+^. As expected, *merA*, *merP*, *merT*, or *merD* could complement the mercury sensitive phenotype of corresponding *P. stutzeri* 273 gene deletion strain (**Figure 2B**). These results clearly demonstrate that *merA*, *merP*, *merT*, and *merD* determine mercury resistance in *P. stutzeri* 273.

The metalloregulatory protein MerR acts as both a repressor and an activator of the transcription of the *mer* operon depending on the absence or presence of mercuric ions, respectively (Champier et al., 2004). MerR is the predominant regulator for mercury resistance, however, the function of the other potential regulator MerD remains speculative. To further clarify the function of MerD and MerR for mercury resistance in *P. stutzeri* 273, we detected the expression of other *mer* genes in the mutant Δ*merR* or Δ*merD* of *P. stutzeri* 273 by qRT-PCR in the presence of different concentrations of Hg^2+^. Interestingly, in the absence of Hg^2+^, the expression of all *mer* genes was up-regulated in the *merR* deletion mutant (**Figure 2C**, left panel), which indicates that merR is a repressor in *P. stutzeri* 273 in the absence of Hg^2+^. However, the expression of all *mer* genes was down-regulated (**Figure 2C**, left panel) in the *merD* deletion mutant, which indicates that merD is an activator in *P. stutzeri* 273 in the absence of Hg^2+^. When challenged with 20 μM Hg^2+^, in the *merR* or *merD* deletion mutant, the expression of most *mer* genes was up-regulated from 4 to 12 times except for *merT* and *merF*, whose expression almost kept the same level when challenged with 20 μM Hg^2+^ (**Figure 2C**, middle panel). Notably, the expression of *merD* or *merR* was up-regulated about eight times in the *merR* or *merD* deletion mutant (**Figure 2C**, middle panel), which suggests that MerD or MerR might regulate the expression of the *mer* gene cluster when the other is absent. In the presence of 50 μM Hg^2+^, the growth of the *merR* deletion mutant was completely inhibited, however, the *merR* deletion mutant grew very well with the expression of *merD* increased more than 60-fold (**Figure 2C**, right panel). Moreover, the expression of all other *mer* genes in *merR* deletion mutant was up-regulated from 20 to 100 times in the presence of 50 μM Hg^2+^ (**Figure 2C**, right panel). Together with the results of gene deletion and complement shown in **Figures 2A,B**, we propose that MerR is a repressor and MerD is an activator in the absence of Hg^2+^, MerD acts as an activator in both low (20 μM) and high (50 μM) concentrations of Hg^2+^, and MerR is only functional as an activator in the presence of low (20 μM) but not high (50 μM) concentration of Hg^2+^.

### Transcriptome Profiles of *P. stutzeri* 273 Challenged With Different Concentrations of Hg^2+^

**FIGURE 3:**
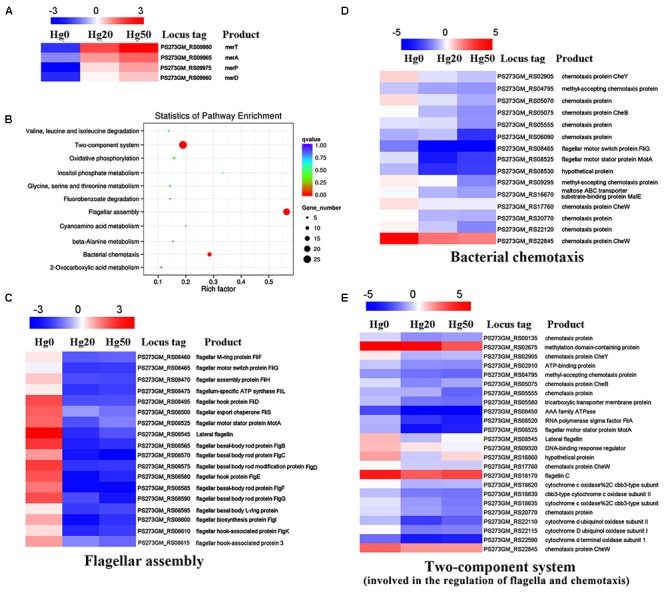
Transcriptome profiles of *P. stutzeri* 273 challenged with different Hg^2+^ concentrations. **(A)** Heat map shows relatively up-regulated and differentially expressed Hg^2+^ stress-responsive genes (DEGs) within *mer* gene cluster in *P. stutzeri* 273 among Hg0, Hg20, and Hg50 enriched by KEGG analysis. **(B)** DEGs enriched KEGG pathway scatterplot of *P. stutzeri* 273 challenged with different Hg^2+^ concentrations. The Rich factor is the ratio of differentially expressed gene numbers annotated in this pathway term to all gene numbers annotated in this pathway term. The greater the Rich factor, the greater the degree of pathway enrichment. A *Q*-value is the corrected *p*-value ranging from 0 to 1, and a lower value indicates greater pathway enrichment. Heat map shows relatively down-regulated Hg^2+^ stress responsive DEGs that contribute to flagellar assembly **(C)**, bacterial chemotaxis **(D)** and two-component system involved in the regulation of flagella and chemotaxis **(E)** among Hg0, Hg20, and Hg50 enriched by KEGG analysis. The locus tag and its corresponding encoding product shown with the heat map. Heat map made using Heml 1.0.3.3.

Mercury and its compounds exert inhibitory effects on the functioning of bacterial enzymes and proteins and render them useless. However, bacterial genetic and morphological flexibility along with immense physiological variability enable them to survive in extreme environmental conditions (Mathema et al., 2011). To reveal the overall responses when exposed to mercury stress, we performed transcriptome analyses of *P. stutzeri* 273 challenged with different concentrations of Hg^2+^. As expected, expression of *merT*, *merA*, *merD*, and *merP* was significantly up-regulated with increasing Hg^2+^ concentrations (**Figure 3A**). These results are consistent with the genetic results (**Figure 2**) and confirm the importance of these genes to confer mercury resistance in *P. stutzeri* 273. KEGG enrichment analysis showed that flagellar assembly, bacterial chemotaxis and two-component system were enriched among the two-way comparison of Hg0 (absence of Hg^2+^), Hg20 (presence of 20 μM Hg^2+^), and Hg50 (presence of 50 μM Hg^2+^) (**Figure 3B**). It is noteworthy that all of the genes expressed related to these three pathways were significantly down-regulated with increasing concentrations of Hg^2+^ (**Figures 3C–E**). There are 18 differentially expressed genes (DEGs), 15 DEGs or 24 DEGs under the terms “flagellar assembly” (**Figure 3C**), “bacterial chemotaxis” (**Figure 3D**) and “two-component system involved in the regulation of flagella and chemotaxis” (**Figure 3E**), respectively. Down-regulation of chemotaxis and two-component system implies that signal transduction of *P. stutzeri* 273 was strictly controlled in response to mercury stress as described previously (Liu et al., 2015). Notably, many down-regulated genes overlapped with different terms, such as PS273GM_RS08465 and PS273GM_RS08525 were classified as both “flagellar assembly” and “bacterial chemotaxis,” and PS273GM_RS08525 belongs to all three pathways, which strongly suggests that these three pathways are closely correlated. We then verified the reliability of the transcriptomic data by qRT-PCR analysis. In total, we selected five genes (*flgG*, *fliC*, *motA*, *cheY*, and *flgB*) that belong to “flagellar assembly,” “bacterial chemotaxis” or “two-component system” for validation. We consistently observed similar trends between qRT-PCR and RNA-seq results, which support the validity of our RNA-seq data (Supplementary Figure S2).

### Different Mercury Concentrations on Physiological Development in *P. stutzeri* 273

**FIGURE 4:**
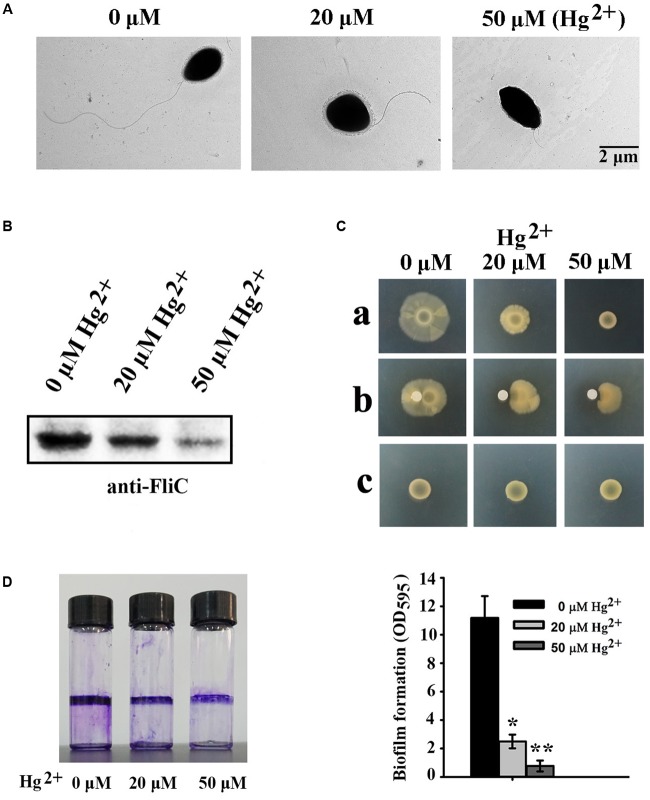
Effects of different Hg^2+^ concentrations on *P. stutzeri* 273 physiology. **(A)** Effects of different Hg^2+^ concentrations on flagella formation in *P. stutzeri* 273 analyzed by TEM. Representative pictures are shown. **(B)** Western blot analysis of protein expression of the typical flagellar related gene *fliC*. **(C)** Effects of different concentrations of Hg^2+^ on motility (row a) and chemotaxis (row b) in *P. stutzeri* 273. Motility assays of *fliC* deletion mutant of *P. stutzeri* 273 in the absence or presence of Hg^2+^ (row c). For the motility assay, the medium contained different Hg^2+^ concentrations. The chemotaxis assay loaded different Hg^2+^ concentrations on the filter paper. **(D)** Effects of different Hg^2+^ concentrations on biofilm formation in *P. stutzeri* 273 analyzed by crystal violet staining (left panel). Quantification shown in right panel.

Because many DEGs involved in “bacterial chemotaxis” or “two-component system” are associated with flagellar formation, we examined the effects of different mercury concentrations on flagella formation in *P. stutzeri* 273 by TEM. Consistent with our transcriptome profile results, flagellar formation in *P. stutzeri* 273 was dramatically inhibited with increased Hg^2+^ concentration (**Figure 4A**). In the condition without mercury stress, we could observe one intact polar flagellum in all cells (**Figure 4A**, left panel). However, most cells formed only abnormal flagella when exposed to Hg^2+^, and the flagellar abnormal ratio and severity were Hg^2+^-concentration dependent (**Figure 4A**, middle and right panels). So, we then examined the protein expression of flagellin (FliC), the typical symbol protein for detecting flagella formation, using western blot (Ammendola et al., 2016). The results showed that FliC expression gradually decreased with increased Hg^2+^ concentration (**Figure 4B**). These results confirm that Hg^2+^ affects bacterial flagella development as shown in **Figure 4A**. Because flagella formation is closely related to bacterial motility, chemotaxis and biofilm formation (Stelmack et al., 1999; Kearns, 2010), we then examined these features of *P. stutzeri* 273 in the absence or presence of Hg^2+^. *P. stutzeri* 273 is a strong motile bacterium, and it swarms in 0.5–1.0% agar plate with a similar speed (Supplementary Figure S3). However, cell motility was significantly inhibited when amended with 20 μM Hg^2+^. This inhibition became more evident when the concentration of Hg^2+^ increased to 50 μM (**Figure 4C**, row a). The bacterial chemotaxis system, which helps bacteria find optimal conditions for growth and survival, decreased toward the Hg^2+^ gradient (**Figure 4C**, row b). Consistent with the result of **Figure 4B**, the bacteria totally lost the motility ability when removing *fliC* from the chromosome regardless the absence or presence of mercury stress (**Figure 4C**, row c). Considering the close relationship among bacterial flagellar formation, motility, chemotaxis, and biofilm formation (Dressaire et al., 2015), we examined biofilm formation of *P. stutzeri* 273 when exposed to Hg^2+^ environment. As expected, the biofilm formation of *P. stutzeri* 273 was dramatically inhibited in the presence of 20 μM or 50 μM Hg^2+^ (**Figure 4D**). This result indicates that *P. stutzeri* 273 physiologically adapted to the mercury stress by down-regulating motility and positive chemotaxis.

### MerF Determines Flagellar Formation and Motility of *P. stutzeri* 273

**FIGURE 5:**
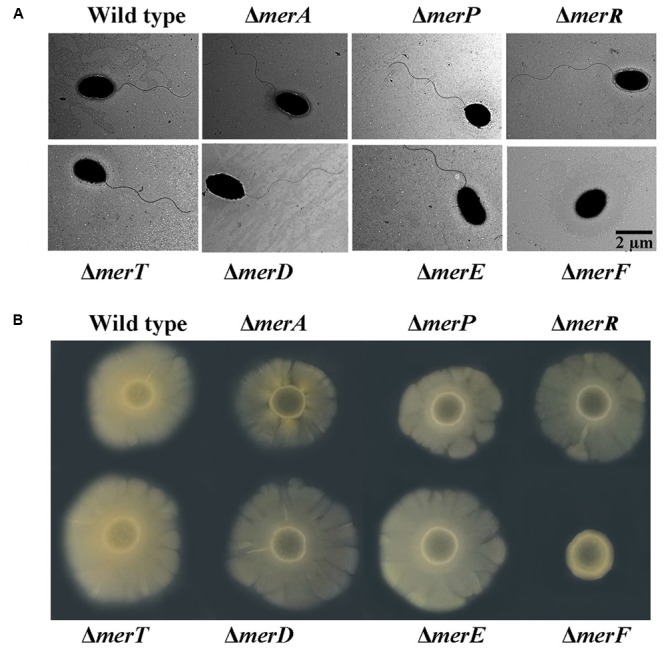
Morphology and motility assays in wild type and different deletion mutants of genes within the *mer* gene cluster. **(A)** Flagellar formation in *P. stutzeri* 273 wild type and mutants (Δ*merA*, Δ*merP*, Δ*merR*, Δ*merT*, Δ*merD*, Δ*merE*, and Δ*merF*) by TEM. **(B)** Motility assays of *P. stutzeri* 273 wild type and mutants (Δ*merA*, Δ*merP*, Δ*merR*, Δ*merT*, Δ*merD*, Δ*merE*, and Δ*merF*) on agar plate.

**FIGURE 6:**
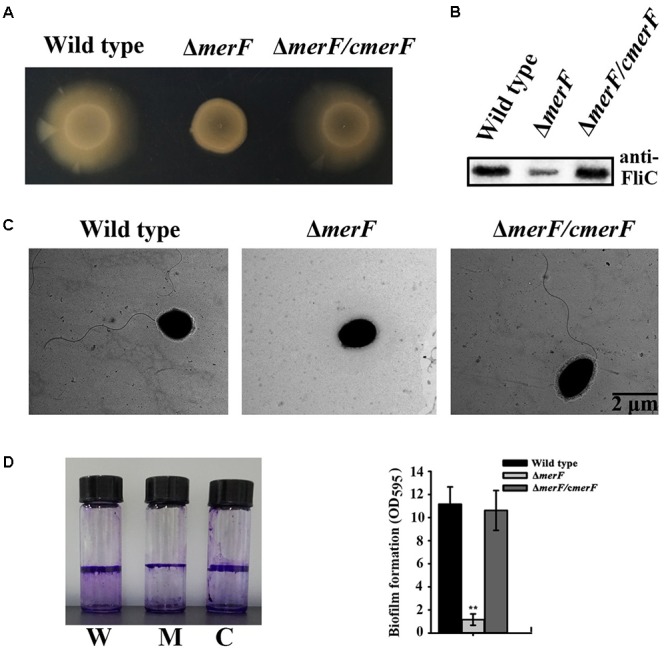
MerF determines flagellar formation and motility of *P. stutzeri* 273. **(A)** Motility assays of *P. stutzeri* 273 wild type, *merF* deletion mutant Δ*merF* and its complementation strain Δ*merF*/*cmerF* on agar plate. **(B)** Western blot analysis of FliC protein expression in *P. stutzeri* 273 wild type, *merF* deletion mutant Δ*merF* and its complementation strain Δ*merF*/*cmerF.* **(C)** Flagella formation in *P. stutzeri* 273 wild type, *merF* deletion mutant Δ*merF* and its complementation strain Δ*merF*/*cmerF.* **(D)** Comparison of biofilm formation in *P. stutzeri* 273 wild type (labeled with W), *merF* deletion mutant Δ*merF* (labeled with M) and its complementation strain Δ*merF*/*cmerF* (labeled with C) by crystal violet staining (left panel). Quantification shown in right panel.

*Mer* operon plays key roles in mercury resistance. But, our results suggest that mercury stress correlates with bacterial physiology. We next examined the possible relationship between *mer* genes and bacterial physiological development. First, we checked the morphology of wild type and *mer*-related gene deletion mutants of *P. stutzeri* 273 via TEM. Surprisingly, the mutant with *merF* deletion (Δ*merF*) could not form flagella compared with wild type and other mutants (**Figure 5A**). The mutant Δ*merF* completely lost motile capability on the agar plate, while wild type and other mutants showed normal motile phenotype (**Figure 5B**). To further elucidate these novel functions of MerF, we constructed a complement of Δ*merF* (Δ*merF/cmerF*). We detected bacterial motility and flagella/biofilm formation. As expected, the complementation of *merF* restored the capabilities of motility (**Figure 6A**), flagella formation (**Figures 6B,C**) and biofilm formation (**Figure 6D**). These results demonstrate that MerF is a key factor to determine flagella formation in *P. stutzeri* 273.

**FIGURE 7:**
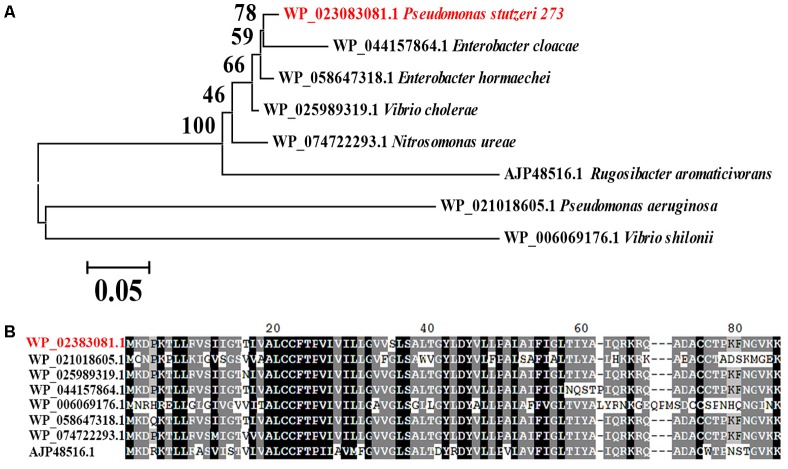
Phylogenetic and conservation analyses of MerF. **(A)** The consensus phylogenetic tree of MerF in *P. stutzeri* 273 with other related MerF obtained from GenBank (accession numbers are indicated before the species name) constructed by the neighbor-joining method. Numbers above the branches are bootstrap values based on 1000 replicates. **(B)** Amino acid sequence alignment of MerF of *P. stutzeri* 273 (WP_023083081.1) with other MerF existing in different pathogenic bacterial strains (WP_021018605.1, *Pseudomonas aeruginosa*; WP_025989319.1, *Vibrio cholera*; WP_044157864.1, *Enterobacter cloacae*; WP_006069176.1, *Vibrio shilonii*; WP_058647318.1, *Enterobacter hormaechei*; WP_074722293.1, *Nitrosomonas ureae*; AJP48516.1, *Rugosibacter aromaticivorans*).

Considering the important role of MerF for flagella development, motility and biofilm formation, we searched for homologs in the NCBI database. Interestingly, MerF homologs of *P. stutzeri* 273 exist in many prevalent pathogens, including some bacterial strains belong to *P. aeruginosa*, *Vibrio cholera*, *Enterobacter cloacae*, *Vibrio shilonii*, and *Enterobacter hormaechei* (**Figure 7A**). Moreover, MerF homologs in these other bacterial strains not only show high identity but also share sveral conserved domains with MerF in *P. stutzeri* 273 (**Figure 7B**).

## Discussion

The extremely broad phenotypic and genotypic diversity of *P. stutzeri* has proven an ideal model system for biochemical characterization of bioremediation and yielded significant advances in this area (Chen et al., 2011). *P. stutzeri* 273 is a marine bacterium isolated from sediments of the East China Sea, an environment existing different heavy metals (Yuan et al., 2004). Bacteria possess an exceptional ability to adapt to their environment, and their genetic and physiological flexibility enables them to develop a variety of survival mechanisms (Mathema et al., 2011). Several gene loci involved in resistance to different heavy metals exist in the genome of *P. stutzeri* 273, such as mercury, cadmium and copper (Wu et al., 2017). Specifically, *P. stutzeri* 273 possesses strong mercury resistance and removal capabilities and there is a *mer* gene cluster located in the chromosome (**Figure 1**). *P. stutzeri* 273 also produces exopolysaccharide EPS273, which effectively inhibited both biofilm formation of *P. aeruginosa* PAO1 and biofouling in the marine environment (Wu et al., 2016). Together with our present discoveries, we deduce that *P. stutzeri* 273 has evolved different strategies to respond to the harsh conditions and compete with other microbes for survival. Overall, we propose *P. stutzeri* 273 will be an ideal candidate to study the adaptation and evolution of marine bacteria to the harsh environment.

In most mercury resistance bacteria, MerR tightly controls the expression of the whole *mer* gene cluster; however, the exact function of the other potential regulatory protein MerD remains unresolved. MerD may function as either an activator (Nucifora et al., 1989) or a repressor of the *mer* operon (Mukhopadhyay et al., 1991). In *P. stutzeri* 273, MerR and MerD were demonstrated to be a repressor and an activator in the absence of Hg^2+^ (**Figure 2C**, left panel), respectively. MerR functions as an activator only in the presence of low concentration of Hg^2+^ (such as 20 μM) (**Figure 2C**, middle panel), however, MerD acts as an activator in both low and high concentrations of Hg^2+^ (**Figure 2C**, middle and right panels). In the presence of mercury stress, MerR and MerD might cooperate to regulate the expression of the *mer* gene cluster as described previously (Champier et al., 2004). We are still not clear the exact action mechanisms of MerR and MerD, which need further elucidation in the future.

Bacteria maintain an uneasy relationship with metal ions and alter their physiological behavior to minimize or nullify the toxicity of metals when residing in such regions or encountering such conditions (Singh et al., 2014). Bacteria can sense metal stress through a receptor and signal(s) using two-component regulatory systems and transmitting information to flagellar motors, which move them in the required direction by the chemotaxis system (Bren and Eisenbach, 2000). Here, we consistently demonstrated using transcriptional profiles that most genes involved in bacterial flagellar assembly, chemotaxis and corresponding two-component system of *P. stutzeri* 273 were dramatically down-regulated when challenged with 50 μM Hg^2+^ (**Figures 3B–D**). Correspondingly, Hg^2+^ significantly altered bacterial physiology development including flagella formation, motility, chemotaxis, and biofilm formation (**Figure 4**).

The question remains why bacteria undergo such physiological responses under mercuric stress. Many metals have no known beneficial function and can be quite toxic, even at low levels. Given our experimental conditions in the presence of Hg^2+^, *P. stutzeri* 273 could only remove mercury near their living microenvironment. However, some amounts of Hg^2+^ still remained in the media, which the bacterial chemotaxis system could sense. Therefore, the optimal bacterial response will decrease motility to avoid more mercuric stress exposure. So, bacteria down-regulate flagella development to reduce motility under these harsh conditions. This is consistent with our finding that flagellum biosynthesis is inhibited by stressful environmental conditions including metal stress due to flagellum biosynthesis which requires significant cellular resources (Soutourina et al., 2002). The results in this study extend our knowledge of the regulatory mechanisms of the bacterial flagellar system and the complex mechanisms governing bacterial motility in response to heavy metal challenges.

While we understand the transformation of toxic Hg^2+^ to non-toxic Hg^0^ mediated by *mer* genes from a genetic perspective, the roles of *mer* genes playing in bacterial physiological development to cope with the mercuric stress remain elusive. Surprisingly, *merF* deletion from the chromosome of *P. stutzeri* 273 led to defective flagella formation (**Figure 5A**) and decreased swarming motility (**Figure 5B**) and biofilm formation (**Figure 6D**), while *merF* complement could restore these disabled functions (**Figures 6A–D**). MerF was reported to have 81 residues, two transmembrane helices and 20% sequence identity with two-thirds of the N-terminal of MerT (116 residues and three transmembrane helices) (Wilson et al., 2000). Previous studies propose that MerF is a mercuric ion transport protein possessing similar functions as MerT and MerC (Wilson et al., 2000). Thus, our findings discovered the connection between the *mer* related gene and bacterial flagella formation. MerF exists in many strains of pathogens, including *P. aeruginosa*, *Vibrio cholera*, and *Enterobacter cloacae*. This finding strongly suggests that MerF homologs in these pathogens may also regulate flagella and biofilm formation. Due to the importance of flagella formation, motility and biofilm formation in pathogenic infection (Chaban et al., 2015; Al-Wrafy et al., 2017), our study provides novel candidates to target some pathogen containing *merF*. Based on the novel MerF function we identified, we hypothesize that *mer* genes have gradually increased their gene complements and functional diversity. We further propose that *mer* evolution has progressed by sequential recruitment of novel functions over evolutionary time (Barkay et al., 2003).

**FIGURE 8:**
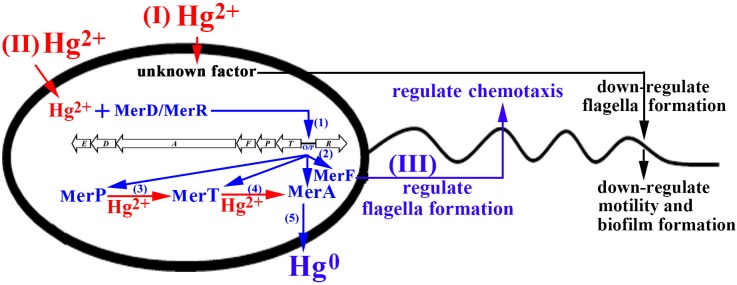
Proposed model of genetic and physiological adaptations of marine bacterium *P. stutzeri* 273 to mercury stress. With the functional *mer* gene cluster in *P. stutzeri* 273, while sensing toxic levels of Hg^2+^, an unknown factor begins to down-regulate flagella/biofilm formation to decrease bacterial motility for passive adaptation to mercury stress (I). (II) Meanwhile, Hg^2+^ enters into the cell and interacts with MerD or MerR to bind *mer* operator/promoter (O/P) region for activating the expression of the *mer* gene cluster (step 1); With the activation of the *mer* gene cluster, MerP/MerT/MerA/MerF are produced in cells (step 2); the Hg^2+^ existing in the cells binds with MerP and then transfers to MerT (step 3). MerT subsequently transfers Hg^2+^ to MerA and converts toxic Hg^2+^ to non-toxic Hg^0^ (step 4). (III) MerF regulates flagella formation and chemotaxis to promote bacterial motility for positive adaptation to mercury stress.

Taken together, we propose a model for the mercury-adapted lifestyle of the marine bacterium *P. stutzeri* 273 which adopts both passive and active strategies to respond to mercury stress (**Figure 8**). We also propose that the *mer* gene cluster in *P. stutzeri* 273 is representative of that found in the genomes of bacteria living in marine environment contaminated by mercury, and this bacterium might be a good candidate to understand bacterial adaptation mechanisms to mercury stress and *mer* genes evolution in marine microbes.

## Author Contributions

RZ and CS conceived and designed the experiments. RZ performed the experiments. RZ, SW, NM, and CS analyzed the data. RZ, SW, and CS wrote the paper.

## Conflict of Interest Statement

The authors declare that the research was conducted in the absence of any commercial or financial relationships that could be construed as a potential conflict of interest.
