# Supplementary material for: Genetic and Physiological Adaptations of Marine Bacterium Pseudomonas stutzeri 273 to Mercury Stress
Source: Front Microbiol. 2018 Apr 5;9:682. doi: 10.3389/fmicb.2018.00682 (PMC5895735; doi:10.3389/fmicb.2018.00682)
Supplement: Supplementary file 1 [file Data_Sheet_1.docx]

**SUPPLEMENTAL MATERIAL**

**Genetic and physiological adaptations of marine bacterium *Pseudomonas stutzeri* 273 to mercury stress**

*Rikuan Zheng^1,2,3^, Shimei Wu^4^, Ning Ma^1,2,3^, Chaomin Sun^1,2 *^*

*^1^Key Laboratory of Experimental Marine Biology, Institute of Oceanology, Chinese Academy of Sciences, Qingdao 266071, China;*

*^2^Laboratory for Marine Biology and Biotechnology, Qingdao National Laboratory for Marine Science and Technology, Qingdao 266071, China;*

*^3^College of Earth Science, University of Chinese Academy of Sciences, Beijing 100049, China;*

*^4^College of Life Sciences, Qingdao University, Qingdao 266071, China.*

*^*^ Corresponding author*

*Chaomin Sun Tel.: +86 532 82898857*

*Fax: +86 532 82898857*

*E-mail address: sunchaomin@qdio.ac.cn*

**Keywords**: marine, *Pseudomonas stutzeri*, mercury, stress, motility, flagella

**Running title:** Adaptations of *P. stutzeri* 273 to mercury stress

**MATERIALS AND METHODS**

**Transcriptional Profiling of *Pseudomonas stutzeri* 273 Challenged with Different Concentrations of Hg^2+^.**

**Library Preparation for Strand-Specific Transcriptome Sequencing**

A total amount of 3 μg RNA per sample was used as input material for the RNA sample preparations. Sequencing libraries were generated using NEBNext^®^ Ultra™ Directional RNA Library Prep Kit for Illumina^®^ (NEB, USA) following manufacturer’s recommendations and index codes were added to attribute sequences to each sample. rRNA is removed using a specialized kit that leaves the mRNA. Fragmentation was carried out using divalent cations under elevated temperature in NEBNext First Strand Synthesis Reaction Buffer（5×）. First strand cDNA was synthesized using random hexamer primer and M-MuLV Reverse Transcriptase（RNaseH^-^）. Second strand cDNA synthesis was subsequently performed using DNA Polymerase I and RNase H. In the reaction buffer, dNTPs with dTTP were replaced by dUTP. Remaining overhangs were converted into blunt ends via exonuclease/polymerase activities. After adenylation of 3’ ends of DNA fragments, NEBNext Adaptor with hairpin loop structure were ligated to prepare for hybridization. In order to select cDNA fragments of preferentially 150~200 bp in length, the library fragments were purified with AMPure XP system (Beckman Coulter, Beverly, USA). Then 3 μL USER Enzyme (NEB，USA) was used with size-selected, adaptor-ligated cDNA at 37 °C for 15 min followed by 5 min at 95 °C before PCR. Then PCR was performed with Phusion High-Fidelity DNA polymerase, Universal PCR primers and Index (X) Primer. At last, products were purified (AMPure XP system) and library quality was assessed on the Agilent Bioanalyzer 2100 system.

1. **Clustering and Sequencing (Novogene Experimental Department).**

The clustering of the index-coded samples was performed on a cBot Cluster Generation System using TruSeq PE Cluster Kit v3-cBot-HS (Illumia) according to the manufacturer’s instructions. After cluster generation, the library preparations were sequenced on an Illumina Hiseq platform and paired-end reads were generated.

1. **Data Analysis**

Raw data (raw reads) of fastq format were firstly processed through in-house perl scripts. In this step, clean data (clean reads) were obtained by removing reads containing adapter, reads containing ploy-N and low quality reads from raw data. At the same time, Q20, Q30 and GC content the clean data were calculated. All the downstream analyses were based on the clean data with high quality. Reference genome and gene model annotation files were downloaded from genome website directly. Both building index of reference genome and aligning clean reads to reference genome were used Bowtie2-2.2.3 ([Langmead and Salzberg, 2012](#_ENREF_3)). HTSeq v0.6.1 was used to count the reads numbers mapped to each gene. And then FPKM of each gene was calculated based on the length of the gene and reads count mapped to this gene. FPKM, expected number of Fragments Per Kilobase of transcript sequence per Millions base pairs sequenced, considers the effect of sequencing depth and gene length for the reads count at the same time, and is currently the most commonly used method for estimating gene expression levels ([Trapnell et al., 2009](#_ENREF_4)).

1. **Differential Expression Analysis**

(For DESeq with biological replicates) Differential expression analysis of two conditions/groups (two biological replicates per condition) was performed using the DESeq R package (1.18.0) ([Anders and Huber, 2010](#_ENREF_1)). DESeq provide statistical routines for determining differential expression in digital gene expression data using a model based on the negative binomial distribution. The resulting *P*-values were adjusted using the Benjamini and Hochberg’s approach for controlling the false discovery rate. Genes with an adjusted *P*-value <0.05 found by DESeq were assigned as differentially expressed. (For DEGSeq without biological replicates) Prior to differential gene expression analysis, for each sequenced library, the read counts were adjusted by edgeR program package through one scaling normalized factor. Differential expression analysis of two conditions was performed using the DEGSeq R package (1.20.0) ([Wang et al., 2010](#_ENREF_5)). The *P* values were adjusted using the Benjamini & Hochberg method. Corrected *P*-value of 0.005 and log2 (Fold change) of 1 were set as the threshold for significantly differential expression.

1. **GO and KEGG Enrichment Analysis of Differentially Expressed Genes**

Gene Ontology (GO) enrichment analysis of differentially expressed genes was implemented by the GOseq R package, in which gene length bias was corrected ([Young et al., 2010](#_ENREF_6)). GO terms with corrected P value less than 0.05 were considered significantly enriched by differential expressed genes. KEGG is a database resource for understanding high-level functions and utilities of the biological system, such as the cell, the organism and the ecosystem, from molecular-level information, especially large-scale molecular datasets generated by genome sequencing and other high-through put experimental technologies (<http://www.genome.jp/kegg/>) ([Kanehisa et al., 2008](#_ENREF_2)). We used KOBAS software to test the statistical enrichment of differential expression genes in KEGG pathways.

**References**

Anders, S., and Huber, W. (2010). Differential expression analysis for sequence count data. *Genome Biol.* 11. doi: Artn R106

10.1186/Gb-2010-11-10-R106

Kanehisa, M., Araki, M., Goto, S., Hattori, M., Hirakawa, M., Itoh, M., et al. (2008). KEGG for linking genomes to life and the environment. *Nucleic Acids Res.* 36**,** D480-D484. doi: 10.1093/nar/gkm882

Langmead, B., and Salzberg, S.L. (2012). Fast gapped-read alignment with Bowtie 2. *Nat. Methods* 9**,** 357-U354. doi: 10.1038/Nmeth.1923

Trapnell, C., Pachter, L., and Salzberg, S.L. (2009). TopHat: discovering splice junctions with RNA-Seq. *Bioinformatics* 25**,** 1105-1111. doi: 10.1093/bioinformatics/btp120

Wang, L.K., Feng, Z.X., Wang, X., Wang, X.W., and Zhang, X.G. (2010). DEGseq: an R package for identifying differentially expressed genes from RNA-seq data. *Bioinformatics* 26**,** 136-138. doi: 10.1093/bioinformatics/btp612

Young, M.D., Wakefield, M.J., Smyth, G.K., and Oshlack, A. (2010). Gene ontology analysis for RNA-seq: accounting for selection bias. *Genome Biol.* 11. doi: Artn R14

10.1186/Gb-2010-11-2-R14

**
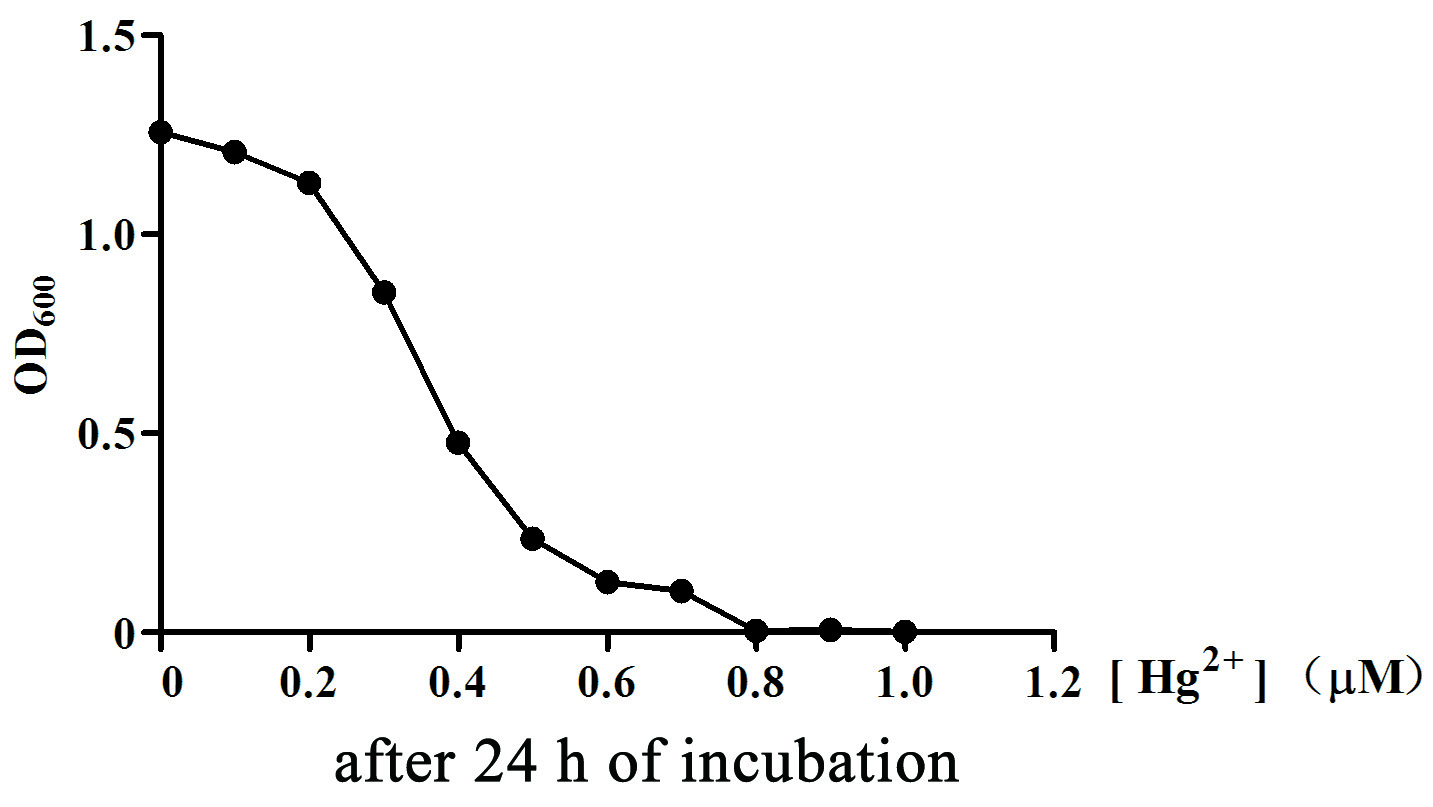
Results**

**Figure s1.** Hg^2+^ MIC determination in *Pseudomonas aeruginosa* PAO1.


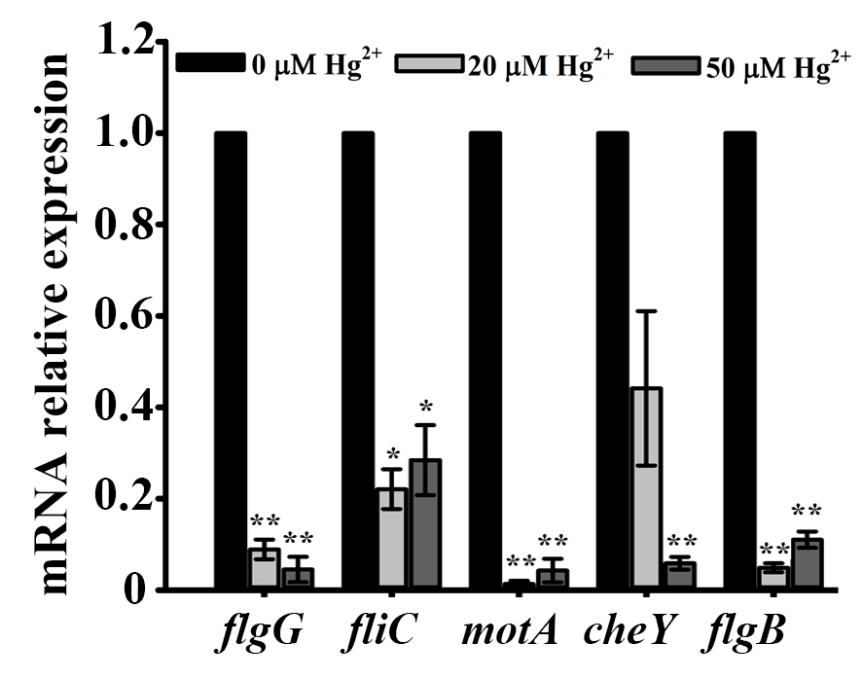
**FIGURE S2.** Gene expression of representative genes related to flagellar assembly, bacterial chemotaxis and two-component system in *P. stutzeri* 273 analyzed by qRT-PCR.

**
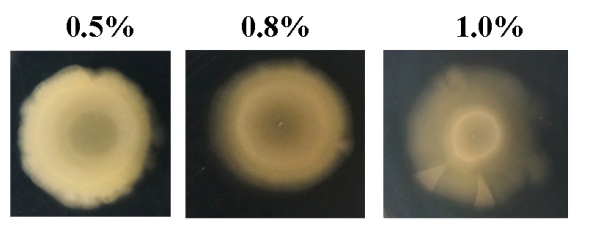
FIGURE S3.** Motility assays of *P. stutzeri* 273 on different concentrations agar

plates.

**Table S1. Primers used in gene knockout**

| Primer name | | Sequence (5’-3’) |
| --- | --- | --- |
| merA-L-f  merA-L-r  merA-R-f  merA-R-r  merR-L-f  merR-L-r  merR-R-f  merR-R-r  merT-L-f  merT-L-r  merT-R-f  merT-R-r  merP-L-f  merP-L-r  merP-R-f  merP-R-r  merF-L-f  merF-L-r  merF-R-f  merF-R-r  merD-L-f  merD-L-r  merD-R-f  merD-R-r  merE-L-f  merE-L-r  merE-R-f  merE-R-r  fliC-L-f  fliC-L-r  fliC-R-f  fliC-R-r | CGCGGATCCGACCAGAAACCGTAGAAGCG (*Bam*HI, underlined)  GCGTCGACCTGGTCAACAAGGGGATGGACT (*Sal*I, underlined) GCGTCGACCGGCTTTCTGGATAGGAC (*Sal*I, underlined) CATGCATGCCGAAAGGACAAACGTATG (*Sph*I, underlined) CCGGAATTCACGAGGGTTGTGCCAATGAT (*Eco*RI, underlined)  CGCGGATCCCATATCGCTTGACTCCGT (*Bam*HI, underlined)  CGCGGATCCATTTCGTGCTATCGCCTTCT (*Bam*HI, underlined)  ATCCTGCAGTTTCATGCCGTAGGGTTC (*Pst*I, underlined)  CCGGAATTCGTCCAGGATGCCTTCGTATT (*Eco*RI, underlined)  CGCGGATCCCTTGCCCTCGCTGCCGTTGT (*Bam*HI, underlined)  CGCGGATCCCGGAGTCAAGCGATATGG (*Bam*HI, underlined)  ATCCTGCAGAACAGGTTGAACACCAGGCGG (*Pst*I, underlined)  CCGGAATTCTCCAGGATGCCTTCGTAT (*Eco*RI, underlined)  CGCGGATCCACCCATCACCAGTCAAGAAC (*Bam*HI, underlined)  CGCGGATCCATGGTGAACTCCTGTGATCAAT (*Bam*HI, underlined)  ATCCTGCAGTAACGAACCGAGCCTGTC (*Pst*I, underlined)  CCGGAATTCAGTTCCAACGCCACCACC (*Eco*RI, underlined)  CGCGGATCCCCCGAAATTCAATGGAG (*Bam*HI, underlined)  CGCGGATCCAGCAGTGTCTTCGGGTCTTT (*Bam*HI, underlined)  ATCCTGCAGCAATCAGCGGGCAGGAAACG (*Pst*I, underlined)  CGCGGATCCTAATGCCCGTTCAGCAGGAT (*Bam*HI, underlined)  ATCCTGCAGAGAGTCTGCCATGAACAGCC (*Pst*I, underlined)  ATCCTGCAGCACGGTGTAGGCGCTCATTG (*Pst*I, underlined)  CCCAAGCTTAATCCAGCCATCACCGTTTT (*Hin*dIII, underlined)  CGCGGATCCGTATGGAACGCAGGTGGG (*Bam*HI, underlined)  ATCCTGCAGTTCCCATTGAGACAAACCAC (*Pst*I, underlined)  ATCCTGCAGGGTTTGTGCGTCTCGG (*Pst*I, underlined)  CCCAAGCTTAGCCAGCCAGGTCGCCCAT (*Hin*dIII, underlined)  CCGGAATTCCGGGGAACCACCTGATCCCATCC (*Eco*RI, underlined)  CGCGGATCCGATGTGCCTCCAAGGGCGCTA (*Bam*HI, underlined)  CGCGGATCCTCGCGAAAAGGCGTTAGCGAT (*Bam*HI, underlined)  ATCCTGCAGGCGTCAGCACTAACCGCGAGCC (*Pst*I, underlined) | |

**Table S2. Primers used in construction of complementary strains**

| Primer name | | Sequence (5’-3’) |
| --- | --- | --- |
| MerA-f  merA-r  merT-f  merT-r  merP-f  merP-r  merD-f  merD-r  merF-f  merF-r | CCCAAGCTTGGGATGACCGAAATCACCGTGAA (*Hin*dIII, underlined)  CGCGGATCCGCGTTATCCAGCGCAGCAGGAAA (*Bam*HI, underlined)  CCCAAGCTTGGGATGTCTGAACCTCAAAACGG (*Hin*dIII, underlined)  CGCGGATCCGCGTCAATAGAAAAATGGCACGA (*Bam*HI, underlined)  CCCAAGCTTGGGATGAAAAAGCTGCTTTCCGC (*Hin*dIII, underlined)  CGCGGATCCGCGTCAGTTCTTGACTGATGATG (*Bam*HI, underlined)  CCCAAGCTTGGGATGAGCGCCTACACCGTGTC (*Hin*dIII, underlined)  CGCGGATCCGCGGTAGCCGGTGATCGGTTTGT (*Bam*HI, underlined)  CCCAAGCTTGGGATGAAAGACCCGAAGACACT (*Hin*dIII, underlined)  CGCGGATCCGCGTCATTTTTTTACTCCATTG (*Bam*HI, underlined) | |

**Table S3. Primers used in qRT-PCR**

| Primer name | Nucleotide Sequence (5’-3’) |
| --- | --- |
| 16s-f  16s-r  merR-f  merR-r  merD-f  merD-r  merA-f  merA-r  merP-f  merP-r  merT-f  merT-r  merE-f  merE-r  merF-f  merF-r  flgG-f  flgG-r  fliC-f  fliC-r  MotA-f  MotA-r  CheY-f  CheY-r  flgB-f  flgB-r | CTTGCTCCATGATTCAGCGG  AGTTACGGATCGTCGCCTTG  TGGAGAACCTGACCATTGGC  CGTTACATCCGTCTCGCCAT  GCACGAAGCACAGCCGTT  GGGTGAGCGTGCATATCGT  GAAGAACAAGGTGCTGCGTG  GCTTGTCAGCGACACCATTC  CATCCTCGGTAGCCTTGGTC  AGGCGATTTCCAAGGTCGAT  GAATCGCGCAAACCTCACC  GATCGGCAACTTGACGGTGT  CGTGTCACAGACAGGACGAAC  CTCACCTGTCCCTGCCATTT  ATGGCGTAGATGGTCAAGCC  CAGCATCATTGGCACAACCC  ACCCTGGTCAACTTCACCAATC  TCATCGCCACCATCGCCTC  TGCCCTCAGCACCTCCAT  TTACGCACGGCCACATTC  GGGTGATTCTTGCCTTGGG  TGTCGTCACGCGGTTGATT  TCAGGATCAGCACCTTCATTTC  ACGACGACCCGACCTTTACT  TACCGATCCGCGTCTGCT  GGCCCTCGATGGCTTGTT |
